# VATS surgical anatomical resection of bronchopulmonary sequestration presenting as chest sepsis

**DOI:** 10.1186/s13019-022-01887-7

**Published:** 2022-05-26

**Authors:** Akshay J. Patel, Tobin Mangel, Rebecca Perris, Islam El-Gamal, Mohamed Shatila, Muhammad Omar Farooq, Maninder S. Kalkat

**Affiliations:** 1 Institute of Immunology and Immunotherapy, University of Birmingham, Birmingham, England, UK; 2 Department of Thoracic Surgery, Queen Elizabeth Hospital, University Hospitals Birmingham, UHB Hospitals NHS Foundation Trust, Mindelsohn Way, Edgbaston, Birmingham, B15 2TH England, UK; 3 Department of Thoracic Surgery, St. George’s Hospital NHS Foundation Trust, London, England, UK

**Keywords:** Intralobar sequestration (ILS), Extralobar sequestration (ELS), Broncho-pulmonary sequestration (BPS), Video assisted thoracoscopic surgery (VATS), Chest sepsis, Pneumonia

## Abstract

**Background:**

Bronchopulmonary sequestration (BPS) is a malformation of the lungs resulting in lung tissue lacking direct communication to the tracheobronchial tree. Most cases demonstrate systemic arterial blood supply from the descending thoracic aorta, the abdominal aorta, celiac axis or splenic artery and venous drainage via the pulmonary veins with occasional drainage into azygos vein. BPS is considered a childhood disease and accounts for 0.15–6.40% of congenital pulmonary malformations. BPS is divided into intralobar sequestrations (ILS) and extralobar sequestrations (ELS) with ILS accounting for 75% of all cases.

**Methods:**

Here we present our 11-year experience of dealing with BPS; all cases presented with recurrent chest sepsis in young-late adulthood regardless of the type of pathological sequestration. The surgical technique employed was a minimally invasive video-assisted thoracoscopic anterior approach (VATS).

**Results:**

Between May 2010 and September 2021, we have operated on nine adult patients with bronchopulmonary sequestration who presented late with symptoms of recurrent chest sepsis. Most patients in the cohort had lower lobe pathology, with a roughly even split between right and left sided pathology. Moreover, the majority were life-long never smokers and an equal preponderance in males and females. The majority were extralobar sequestrations (56%) with pathological features in keeping with extensive bronchopneumonia and bronchiectasis. There were no major intra-operative or indeed post-operative complications. Median length of stay was 3 days.

**Conclusions:**

Dissection and division of the systemic feeding vessel was readily achievable through a successful anterior VATS approach, regardless of the type of sequestration and without the use of pre-operative coiling of embolization techniques. This approach gave excellent access to the hilar structures yet in this pathology, judicious and perhaps a lower threshold for open approach should be considered.

## Introduction

Bronchopulmonary sequestration (BPS) is a mass of abnormal lung tissue usually of embryonic origin from the caudal foregut that does not possess an anatomical connection to the tracheobronchial tree. Generally speaking, pulmonary sequestrations are of two types: intralobar (ILS) and extralobar (ELS), with the majority being ILS, circa 85% [1]. ELS are usually separated from the normal lung parenchyma but its own visceral pleura, whereas ILS is incorporated within normal lung tissue. Furthermore, pulmonary sequestration is vascularised by an aberrant systemic artery that most commonly arises from the descending thoracic or abdominal aorta [2]. In the case of ILS, venous drainage is via the pulmonary vein whereas with ELS this can occur via the pulmonary or systemic venous system [2]. Symptomatic cases of sequestration are usually dealt with by surgical resection either anatomical or sub-lobar resection. Formerly, the approach for such operations was through a postero-lateral thoracotomy, however in the era of minimally invasive surgery, increasing use of video assisted thoracoscopic surgery (VATS) and robotic approaches have been reported [3–5].

The difficulty with the VATS approach resides with the risk of injury to the systemic feeding vessel to the sequestered lung. Previous groups [3] have reported pre-operative embolization of said feeding vessel prior to formal resection. Here we report our 11-year experience of the VATS approach in the anatomical resection of bronchopulmonary sequestration.

## Materials and methods

Between May 2010 and September 2021, we have operated on nine adult patients with bronchopulmonary sequestration who presented late with symptoms of recurrent chest sepsis. Case notes were retrospectively reviewed and here we present our single-centre experience.

### Surgical technique

**Fig. 1:**
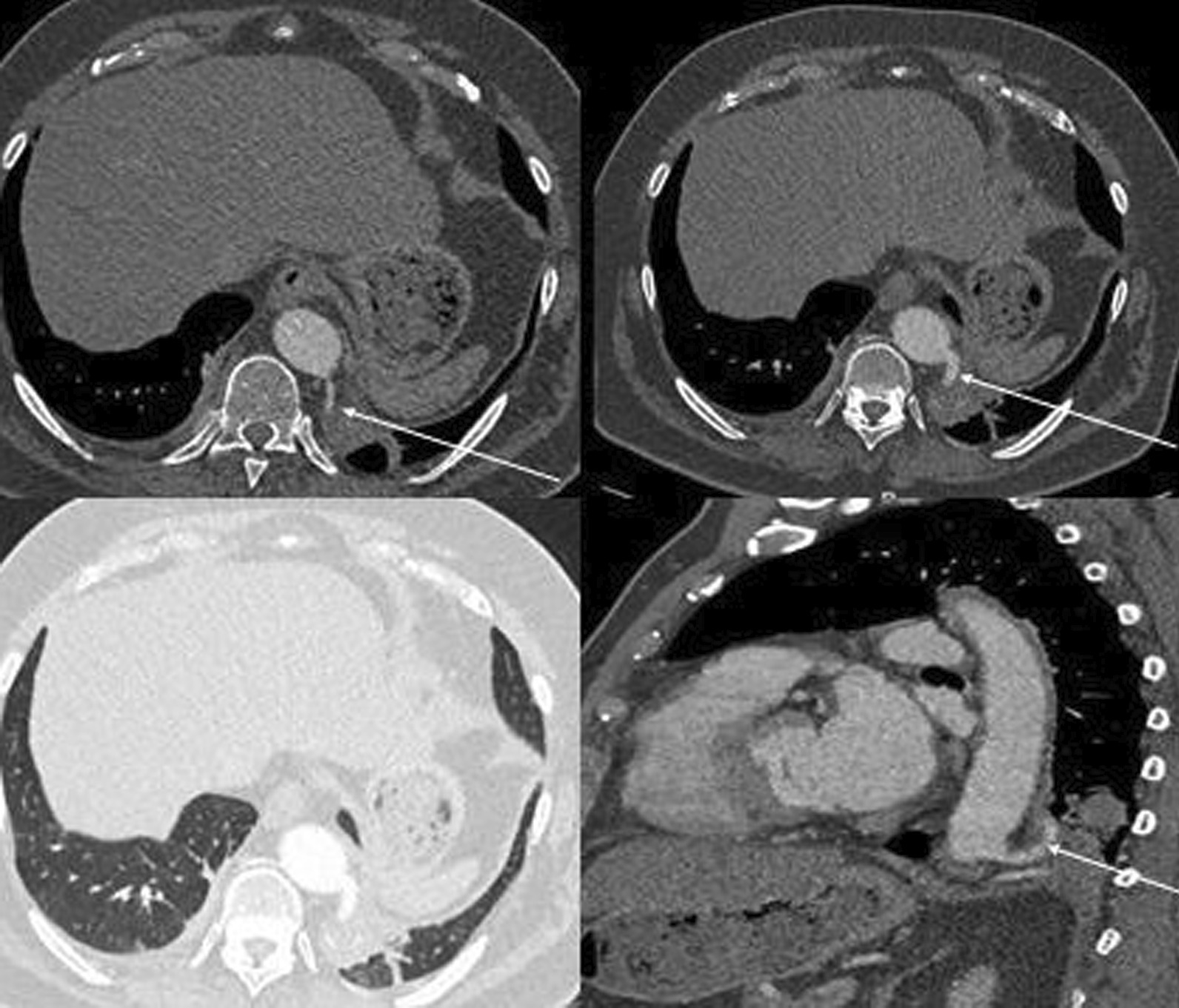
CT slices to illustrate feeding vessels from the retro-aortic position with posterolateral take-off (demarcated with arrows)

Surgical planning involves careful analysis of the computed tomography (CT) scan to ascertain the location of the systemic feeding vessel and its relationship to the sequestered lobe (Fig. 1). Figure 1 demonstrates the two feeding vessels to the lobe and the relevant take-off from the main arterial supply. For the surgical procedure, the patients undergo a general anaesthetic with subsequent rigid bronchoscopy prior to double-lumen tube endotracheal intubation; the non-operative side is solely ventilated.

For left-sided resections, the patient is placed in the right lateral decubitus position and vice-versa. The preparation of the skin and the draping of the patient are done as it would be for a postero-lateral thoracotomy.

An anterior VATS approach is the preferred method; 3 anterior ports are placed as follows, the first port is placed in 5th intercostal space lateral to mid-clavicular line (this is the sub-mammary incision and is known as the universal port), the second port is placed postero-inferior to first port in 7th intercostal space and the third port is placed in the 4th intercostal space in the mid-axillary line. The camera is usually placed in the inferior most port (i.e. the second port).

**Fig. 2:**
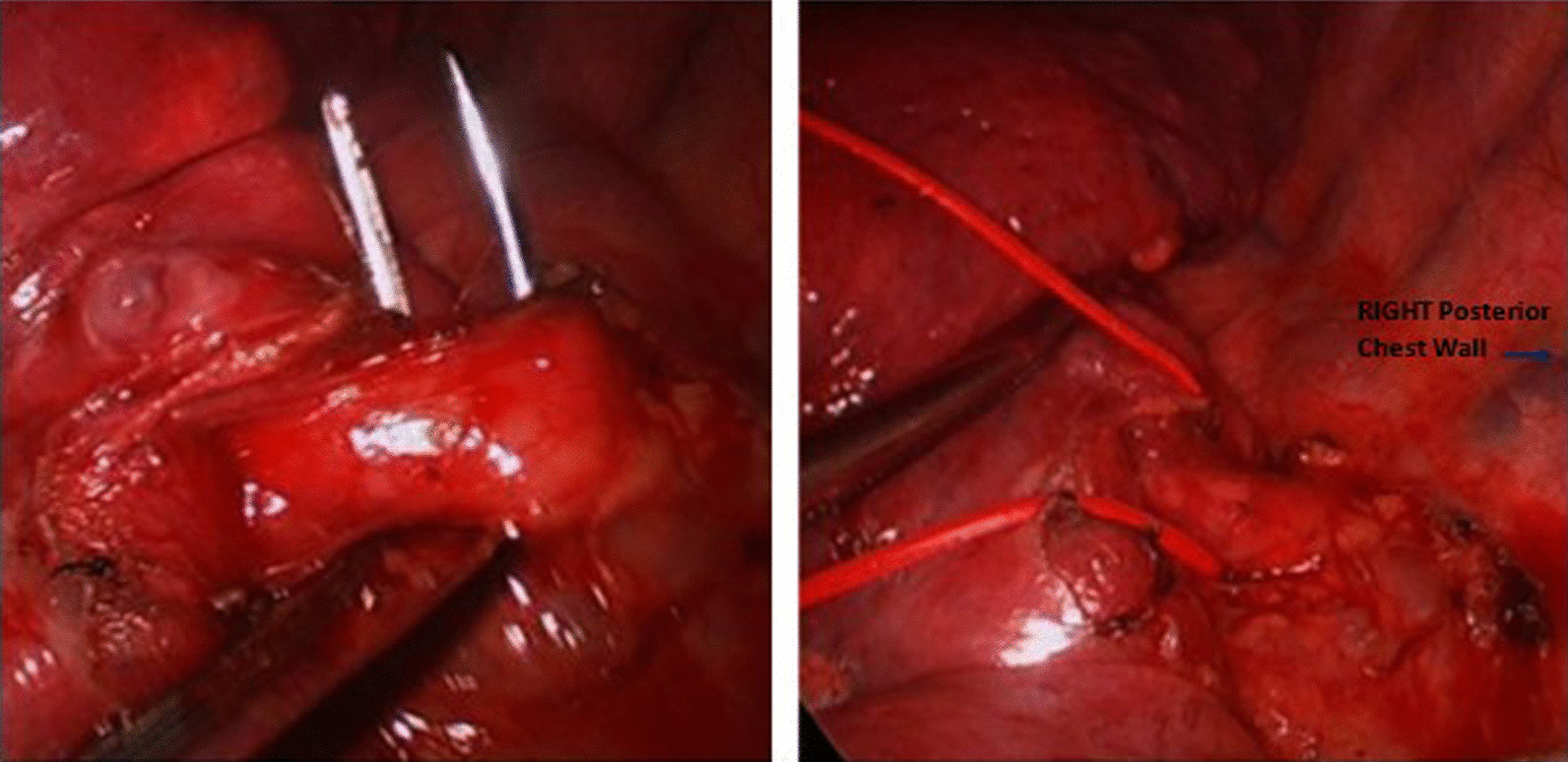
Left sided VATS approach: aberrant direct aortic feeding vessel dissected out using hook diathermy and retro-posteriorly passed using right-angled instrument (left-hand panel). L sided VATS approach: aberrant aortic feeding vessel encircled using rubber sloup in preparation for division with vascular stapler (right-hand panel)

On entry into the chest, in the case of intra-lobar sequestration the pleural space, fissure definition and lobar integrity are assessed. The attention is then drawn to the inferior pulmonary ligament which is carefully dissected using hook electro-cautery. This manoeuvre brings the aberrant systemic arterial vessels from the descending aorta into view (Fig. 2). The vessels are dissected out and then divided separately using an EndoGIA with tristaple technology stapling device with vascular (tan) reloads. A vascular clamp is positioned without closing on the proximal side of the proposed staple line, as a precaution in the event of bleeding. The lobar resection proceeds in the conventional fashion, sequentially dividing the pulmonary vessels and the related bronchus. In one case, the degree of anatomical invasion of the abnormal lung tissue was appropriate enough to warrant a wedge resection.

**Fig. 3:**
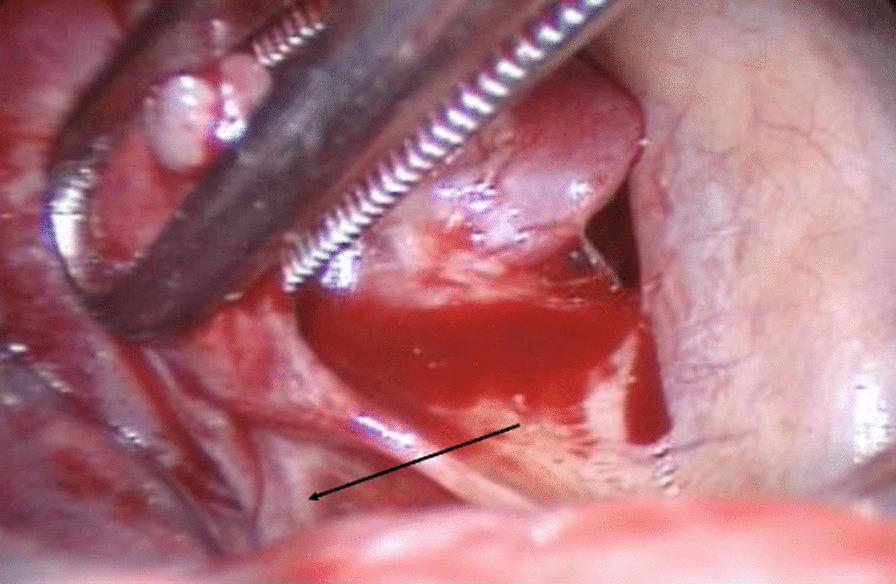
Intra-operative image illustrating the extra-lobar sequestration with direct feeding from an aberrant intercostal artery (black arrow)

In the case of extralobar sequestration, the systemic feeding vessels were carefully dissected and divided using the vascular staplers as described above. In an unusual case in this series, the extralobar sequestration was in relation to the right upper lobe with the feeding vessels apparently coming off the intercostal pedicle posteriorly (Fig. 3). The sequestrated segment was separated from rest of the lobe aided by firing of the stapler across any parenchymal or fibrous connection.

Surgical removal of the sequestrated lung should avoid excessive compression of lung tissue, which can cause abscess embolization via the venous drainage channels and lead to systemic sepsis. Where possible, during the operation, we adhered to dividing the vein following the aberrant arterial division to mitigate against the risk of the above.

## Results

At our institution nine cases with bronchopulmonary sequestration have been operated upon. The demographic, pre-operative and pathological characteristics of our cohort are shown below in Table 1.

**Table 1:** Patient characteristics (n = 9)

Age (years) [median, (IQR)]	34.5, [29–50]
% Male	67% (n = 6)
Laterality (% L)	44% (n = 4)
Laterality (% R)	56% (n = 5)
VATS approach	100%
% Lower Lobe	78% (n = 7)
Mean % DLco	100%
% Never smokers	89% (n = 8)
Average PS	0 (n = 9)
Mean operative time	155 ± 16 min (n = 9)
Mean intra-operative blood loss	320 ± 43mls (n = 9)
Conversion to open	0
Presence of dense adhesions overlying feeding vessel	33% (n = 3)
Pleural plaques	0
Mean Length of post-operative stay (days)	3
Pathology	Intralobar sequestration (n = 4)
	Extralobar sequestration (n = 5)
Presenting complaint	Recurrent Chest Infections (n = 9)

Most patients in the cohort had lower lobe pathology, with a roughly even split between right and left sided pathology. Moreover, the majority were life-long never smokers and an equal preponderance in males and females. The majority were extralobar sequestrations (56%) with pathological features in keeping with extensive bronchopneumonia and bronchiectasis.

**Fig. 4:**
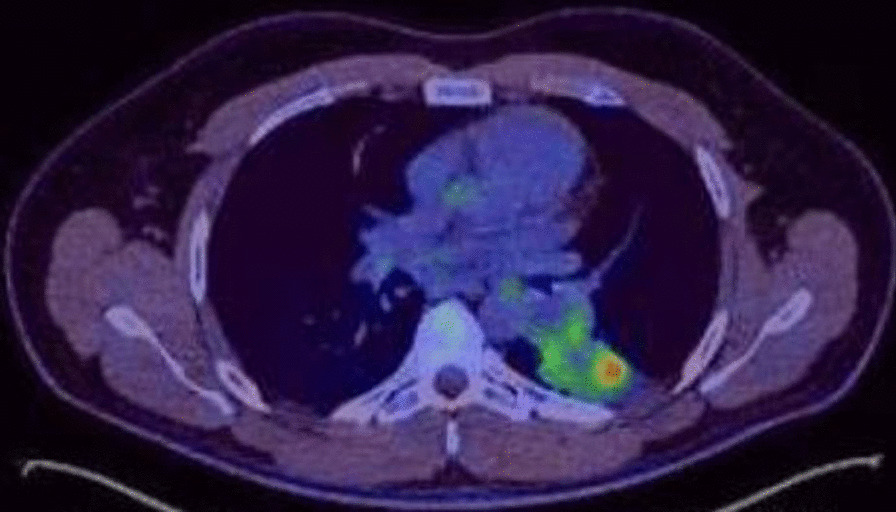
Pre-operative PET-CT slice to correlate findings from Fig. 2. Within this consolidation, there is impression of a rounded abnormality on the PET component showing nodular peripheral activity (SUV Max 8.7) with central inactivity. This measures approximately 3.4 cm × 3.3 cm. The remainder of the consolidation shows no significant activity

All patients had a history of recurrent chest sepsis and underwent pre-operative investigations to include Chest X-ray (CXR) and a CT thorax. In cases where there were mass lesions identified within areas of consolidation, Positron emission tomography-CT (PET-CT) was employed to corroborate findings and to rule out the distant spread of disease in the event of an underlying neoplastic process (Fig. 4). Bronchoscopic evaluation and tissue sampling confirmed inflammatory and possible granulomatous material. There was no history of immunosuppression, and no significant metabolic activity was noted elsewhere.

### Intra-operative process

The primary intra-operative parameters are detailed in Table 1. Owing to the risk of catastrophic bleeding, extreme care was taken when dissecting the aberrant feeder vessel which meant a slightly longer mean operative time of 155 min, but average blood loss was less than half a litre. There were no conversions to open, despite the presence of dense adhesions overlying the feeder vessel in one third of patients. The presence of these adhesions in the paravertebral gutter usually meant routine employment of an energy device such as a harmonic scalpel to avoid excessive blood loss when dissecting the vena comitantes in the peri-vascular fat.

### Post-operative follow-up

Six out of nine patients presented with normal baseline inflammatory markers despite respiratory symptoms. One third of patients, presented with more severe productive symptoms with raised baseline inflammatory markers (CRP > 20, WCC > 12), following surgery once the stress response had settled, there was clinical and biochemical improvement in all patient parameters without the need for aggressive long-term anti-microbial therapy. All patients received prophylactic Co-amoxiclav or Gentamicin/Metronidazole (in the case of penicillin allergy) at induction. Further treatment was directed according to sensitivities and clinical suspicion. There was no incidence of delayed bleeding, pleural effusion, chylothorax, arrhythmia or superimposed pulmonary sepsis in the post-operative period. One case experienced intractable air leak which settled over 3 weeks with flutter bag.

At long-term follow-up (Median 4.2 years [3.2–5.6 years]), all patients were symptom free with no recurrence of productive respiratory infections. All follow-up was conducted clinically with serial CXR in the outpatient department which showed good resolution of the radiographic picture when compared to pre-operatively.

### Comment

The reported patient population is small, however multiple case series exist which report on the feasibility and safety of VATS lobectomy for BPS in experienced hands [3–5]. Most patients with BPS have been shown to present with haemoptysis or recurrent chest sepsis, symptoms that are suggestive of bronchiectasis; certain groups have thus suggested that BPS in adults should be nosologically very similar to acquired lesions such as bronchiectasis [4]. Classically, BPS is a disease of childhood presentation, with ILS presenting more in adulthood often as recurrent chest sepsis. However, a key difference between ELS and ILS is presentation, with ELS presenting early in life and patients having recurrent respiratory infections, respiratory distress, and congestive heart failure. However, most of our series was pathologically identified as having ELS and these patients presented much later in life.

Spiral CT scanning with contrast angiography has been heralded as the gold standard diagnostic strategy for BPS [5]. Spontaneous involution of such lesions [6] and conservative management with arterial embolization have been reported, however definitive treatment is always surgical [5].

Kestenholz and Liu’s groups have reported on the thoracoscopic management of pulmonary sequestration. Both groups state that the difficulty lies in the identification and dissection of the aberrant systemic feeding artery, which can often be buried in dense adhesions that can make identification within the inferior pulmonary ligament difficult [2, 7]. In this group of patients, VATS is readily feasible unless there are adhesions which are too dense for safe dissection and hence one disadvantage is having the lower threshold to convert to an open operation. We did not convert any of our cases, however with the beauty of retrospect, a decision to convert may have reduced operative times and in higher risk patients with poor PS and baseline lung function, a shorter anaesthetic time is of benefit. Groups have stated the advantage of VATS over open in general thoracic surgeon, particularly in light of the VIOLET trial findings and whilst this still holds true in this unique group of patients, one must have a lower threshold to open in the presence of thoracic cavitating lesions in the sequestered lung and dense adhesions over the hilum when the aberrant feeder vessel cannot be readily identified [2, 7].

Since Wan et al. [8] first described the VATS approach for managing BPS in 2002; it has become widely adopted as the approach of choice. A comparative retrospective analysis [7] showed no difference between the open and VATS approach for BPS surgical resection both in terms of operative duration, length of post-operative stays and complications. Treatment of ILS has been thought to be more challenging than for ELS owing to the more challenging anatomical or near-anatomical resection [9]. Wei’s analysis of 2625 BPS patients concluded that timely surgical treatment should be expedited for patients, particularly those presenting with recurrent chest sepsis or in cases where cancer cannot be excluded [10, 11].

Exposing and isolating the aberrant feeding vessel does come with a high risk of bleeding; Zhang’s retrospective series showed that in 21 cases of BPS that were treated surgically, 2 patients suffered massive intra-operative haemorrhage [12]. Comparative analysis with 7 patients who underwent endovascular intervention for BPS, showed no cases of bleeding but instead post-embolisation syndrome (fever and pain at embolism site) in 2 patients [12].

Endovascular occlusion with embolizing agents or microcoils can minimize intraoperative bleeding by reducing blood flow and causing necrosis to occur. Groups have reported successful endovascular occlusion of large aberrant atherosclerotic aortic feeding vessels to minimise intra-operative bleeding at the time of successful uniportal VATS lobectomy [13]. Furthermore, this hybrid approach has been used successfully in 3 patients with surgery taking place 24 h post embolization [14]. The embolization did result in intense thoracic pain in all patients in this series [14]. Endovascular treatment alone has high recurrence rates and may put the patient at risk for developing recurrent infections, abscesses, and haemoptysis [3, 15]. In our series, the pre-operative vessel coiling, or embolization was not performed. We anticipated the resultant necrosis and inflammation will make the dissection and isolation of the feeding pedicle difficult. Furthermore, the material used for the embolization could interfere with the safe application of the vascular staplers, unless proximal placement of such material could be ensured. We demonstrated that the VATS approach is a safe and effective method to remove the sequestered portion of lung. Future management can consider a hybrid approach using both endovascular occlusion with minimally invasive surgery in appropriately selected cases but is by no means a requisite.

## Data Availability

All data and materials are available upon reasonable request from the corresponding author.
